# The British Journals, &c.: Pathology, Practical Medicine, and Therapeutics

**Published:** 1842-04

**Authors:** 


### PATHOLOGY, PRACTICAL MEDICINE, AND THERAPEUTICS.

Partial Hypertrophy of the Heart. The Hon. Mr. R——, about 40 years of age, came under my observation some five or six weeks before his death. He was greatly emaciated—sallow in his complexion—and with extremely feeble circulation. The pulse was like a thread at the wrist, and the action of the heart was scarcely audible. The appetite was nearly wanting, and as food produced distressing feelings, he had long adopted a very rigid and abstemious diet, which probably tended to increase the emaciation. I examined him with the greatest care from head to foot, without being able to detect any lesion that could account for the progressive waste of flesh. The patient (if he could be called such) went direct to Leamington, and never took anything which I prescribed. He placed himself under Dr. Jephson, who gave favorable hopes to his family of ultimate recovery. A month's treatment, however, produced no amendment, and Mr. R. returned, with great difficulty, and in the most exhausted state to London, when he came again under my care. The emaciation was now at its utmost extreme, and as he could take very little nourishment by the mouth, he was supported by strong beaf-tea thrown up into the colon every eight hours. The pulse was now scarcely perceptible at the wrist, and the action of the heart inaudible in any part of the chest. He had no cough—clean tongue—intellects unclouded—and, after lingering about a week, he ceased to exist—pale and pulseless, like a person expiring of cholera

On dissection (by my son) twenty-four hours after death, all the organs and structures in the body, with one exception, were sound. On opening the chest, the lungs, on both sides, completely collapsed, leaving in view a heart of the most extraordinarily small dimensions. The pericardium was intimately glued to the heart, so as to be incapable of separation, so ancient and complete had been the adhesion. The heart itself was not more than half its natural size; yet its parietes (ventricular) were full an inch in thickness. This diminution of general size, with hypertrophy of the walls, had so encroached upon the cavities, that the left chamber could not contain more than three teaspoonfuls of blood! The consequence was, that at each ventricular contraction about one fourth of the normal or natural quantity of blood was discharged from the heart into both aortic and pulmonary system. The general emaciation of the whole body thus so very imperfectly supplied with the vital fluid, is readily accounted for. In respect to diagnosis during life, I conceive that in a case of this kind, and to this extent, it would require more diagnostic means than we yet possess to detect the true nature of the organic lesion.—Dr. James Johnson, *Medico-Chirurgical Review*, No. lxxi. *Jan.* 1842.

---

Abscesses of the Lungs The following remarks (which are illustrated by several striking cases) are worthy of attention :

Though the introduction of the stethoscope has been of the greatest utility in the investigation of pulmonary complaints, both as regards their prognosis and their treatment, it must be confessed that, in many instances, practitioners have been induced unduly to rely upon the indications of disease which this instrument affords, and consequently have seen their prognosis fail. The following remarkable cases afford abundant proof, that patients may recover, contrary to the usual interpretation of the most significant and decisive stethoscopic symptoms, and therefore seem to merit publication, in order to warn practitioners from relying too exclusively upon physical phenomena, and too hastily concluding that pulmonary lesions, however extensive, thus indicated, must necessarily prove fatal. These cases, too, show that vast abscesses may be formed in the lungs, and yet the patient recover; and likewise, that real circumscribed abscess occurs more frequently in the pulmonary tissue than Laennec allowed, or his followers seem to believe. It is true, indeed, that where suppuration takes place in the lung, nature effects it in a manner either calculated to afford the readiest exit for the matter so formed, or best suited to promote its absorption.

This object, from the extent of the parenchymatous structure of these organs, and its relation to the air-cells and minute bronchial tubes, is most easily effected, by so disposing of the purulent fluid, resulting from inflammation, that it can, on the one hand, be with facility eliminated through the bronchial tubes, or on the other absorbed in the texture of the lung itself. In other organs and other parts, a similar facility for mechanical elimination does not exist, and consequently the easiest step which nature can take is, to collect the puriform fluid, within the parietes of a circumscribed abscess, which may work its way outwards for the purposes of discharge. From this view it appears, that in other parts, circumscribed abscess is the ordinary means of evacuation provided by nature, and diffuse suppuration the exception; while in the lungs the reverse obtains, diffuse suppuration being the ordinary rule, and circumscribed abscess the exception. The rationale here exposed has been well explained by Dr. Stokes, in his admirable treatise on diseases of the lungs, but at the time he wrote, neither he nor I was aware that large abscesses occur so frequently in the lungs, or are so often recovered from, as subsequent observation has shown to occur.—Dr. Graves, *Dublin Journal of Medical Science*, No. lx. *Jan.* 1842.

---

Pyrosis from an Elongated and Depressed Ensiform Cartilage. The subject of this case, a man 39 years of age, had observed that he obtained relief from the pyrotic symptoms, on loosening the waist-belt or strap with which he supported his trousers. On examination, the ensiform cartilage was found exceedingly long and incurved. The man was not only aware of this peculiarity, but also of its influence in exciting his disease—in consequence of which, he was accustomed to bring the upper edge of the strap under the cartilage, which he could readily do, from his great emaciation. From frequent repetitions of this manœuvre, he had at length produced a complete dislocation of the cartilage, which could be felt lying loose in the epigastrium. His disease continues; and the reporter of the case is of opinion, that while the peculiar conformation of the ensiform cartilage was the original cause of the pyrosis, this affection is now independent, and will issue in structural change.—Dr. Bird, *Medical Gazette*, No. xi. *Dec.* 3, 1841.

---

Seat and Nature of Favus. Baudelocque supposed that the favous matter is deposited *under* the cuticle reflected into the hair follicle. Rayer believes that it is deposited *between* the hair and the cuticle. The author adopts the latter view, being satisfied that the favous secretion takes place *within* the follicle, but *upon* the cuticle lining it. So much for the *seat* of favus. As to the nature of it, he is of opinion that the matter of favus is a modification of tubercle; that it does not resemble lupus, which consists of small firm tumours; but that it is a disease, the essential characteristic of which is the deposition of that heterologous formation called tubercle.

Favus differs from pustular disease, with which it is often confounded, both in its elementary form, and in the qualities of the matter secreted. Favus is poured out on a free surface, not upon or within the cutis, and under the cuticle; which is always the case with pustule. Favus, in contradistinction to pustule, is chronic, and differs from pus in its solidifying immediately and independent of exposure to the air: it also differs in its chemical constitution.

On the other hand, favus agrees with tubercle, in the circumstance of its quickly solidifying; in its growing by opposition, not intus-susception; in its tendency to assume an ovoid form: in its yellowish opaque white or grayish colour; in its chemical constitution; in the causes producing it, as per diet, and in its being hereditary.—Mr. ERICHSEN, *Medical Gazette*, No. xii. *Dec.* 10, 1841.

---

BLOODLETTING IN MANIA. The object of this paper is to prove the extreme rarity of the cases in which sanguineous depletion is justifiable in mania. The author observes, it may be asked, in what case is the abstraction of blood admissible? With regard to general bloodletting, I repeat that it is very rarely indeed that it can be either indicated or justified. Most of the cases, I believe, in which it has been tried with beneficial results, would, were we in possession of sufficient details, turn out to be cases in which there was inflammation of the brain or its membranes, of a more or less acute character; cases, in short, of encephalitis, in which some of the symptoms were perhaps slightly modified by accidental circumstances or individual peculiarities. Such cases occasionally find their way within the walls of institutions devoted to the treatment of the insane, and by the hasty or inexperienced observer may readily be mistaken for acute mania. There are, however, certain cases, both of mania and other forms of insanity, in which there is an evident congestion of the vessels of the head; where the pulse is firm; the pupil contracted; where everything leads us to believe that the congestion is of an *active* not a *passive* character; where there is no reason to fear a want of vascular tone or apprehend effusion; and in such cases the judicious application of leeches or the cupping-glasses is calculated to be highly beneficial. Even when the congestion is of a more passive character, and where the veins of the scalp are full, tumid, and tortuous, a very moderate and cautious employment of local bloodletting may be serviceable; although in this last case the safer expedient of dry cupping on the nape of the neck, and along the course of the spine, will be frequently found to answer the end as well.—Dr. CRAWFORD, *Medical Gazette*, No. xv. *Dec.* 31, 1841.

---

VACCINE VIRUS. The author of this paper thus explains the fact of persons who have been vaccinated being attacked by smallpox.

The deterioration of the virus, I believe, never is the cause of failure. I have found that the want of success arises most frequently from operating before the pock is perfectly matured. Out of one hundred cases, I vaccinated fifty on the eighth day, and the remainder on the ninth. Of the first half I had to repeat the operation twice in seven cases, while in those done on the ninth day I had only to repeat it in one case: than which no stronger proof can be had of the advantage of having the virus of a proper consistence (not watery) before it is used —Mr. JOHN PATERSON, *Medical Gazette*, No. xv. *Dec.* 31, 1841.

---

IDIOPATHIC HYDROPHOBIA. A man, 24 years of age, subject to cardiac palpitations, having ridden fourteen miles, was seized with sudden dyspnœa, accompanied with pain at the region of the heart and in the chest. After a few minutes, these symptoms subsided, and the patient ate heartily at dinner, and was again attacked in the way described in the evening. He was now bled, first to the extent of ℥xviij, and soon after to ℥vj. The last bloodletting relieved him. The author of this paper saw him at five o'clock on the following morning, speechless, though conscious: convulsively tossing his arms: with a spasmodic constriction of the glottis and pharynx. He had at this time a great desire for water; but no sooner was this presented to him, than an alarming increase of pain about the pharnyx, with a dreadful feeling of suffocation supervened. These immediately left him on the water being withdrawn. The author, struck with these symptoms, questioned him closely whether he had been exposed to hydrophobic contagion: and the patient assured him he had not. Tenderness was detected at the lower part of the cervical region pressure at which point reproduced all the symptoms above described. A blister was then applied, which restored deglutition; the patient, after some further treatment, recovered.—Mr. KIMBLE, *Provincial Medical and Surgical Journal*, No. xv. *Jan.* 8, 1842.

Quinine in Intermittent and Remittent Fever. The author, if he sees the patient sufficiently early in the disease, invariably bleeds in the first stage, after which he places ten or fifteen cupping-glasses over the stomach, withholds all medicine; directs ice to be eaten *ad libitum;* and the patient's hands to be kept in ice. By these means, he tells us, he generally succeeds in converting the remittent into an intermittent, thus affording a more favorable opportunity for the administration of quinine. This substance he then orders in doses of ten or twenty grains; never troubling himself about previously unloading the bowels of the patient. When *tinnitus aurium*, which, according to him, characterizes the full effect of quinine, is perceived, he confidently expects a successful issue. The quinine he prefers giving in solution with tartaric acid; adding the latter to a solution of the former, in a tablespoonful of water, till the water becomes clear.—Dr Petherbridge, *Maryland Medical and Surgical Journal*, No. i. *July*, 1841.

---

Quinine in Agues. This paper, by another writer and in another American Journal, confirming the statements of the one immediately preceding, we quote the following numerical details:

*Of the doses of medicine exhibited on the first day after being received on the sick report, and the character of the disease determined.*—Five grains repeated in twenty minutes in *one* case; five grains repeated in thirty minutes in *one* case; ten grains repeated in thirty minutes in *one* case; ten grains repeated in four hours in *one* case; five grains morning, noon, and evening in *three* cases; eight grains morning, noon, and evening in *one* case; five grains morning, noon, and evening in *one* case; twenty grains morning, noon, and evening in *nine* cases; twenty-five grains morning, noon, and evening in *five* cases; fifteen grains morning, noon, and evening in *four* cases; forty grains morning, noon, and evening, *one* case. Number of cases twenty-eight.

*Of the immediate effects of the medicine.*—No unpleasant effects worthy of record followed the administration in *nineteen* cases. Nausea occurred, apparently in consequence, in *four* cases; vomiting in *one* case; headach in *four* cases; temporary deafness in *four* cases; giddiness, or dizziness, in *nine* cases; buzzing, or ringing in the ears, in *eight* cases.

*Effects of the paroxysms.*—In *nine* cases no paroxysm occurred after the first day of the administration of the specific; in *eleven* cases one subsequent paroxysm only occurred; in *five* cases two only occurred; in *one* case three occurred; in *one* four occurred, the last of which is recorded extremely light; in no case was the treatment unsuccessful.—Dr. Flint, *American Journal of Medical Science*, No. iv. *Oct.* 1841.

---

Morbus Brightii. Is there such a Disease? The author devotes part of a paper "on the treatment of various diseases," to show that Rayer, in his recent work on albuminuria, not only contradicts himself, but admits that there is nothing absolutely distinctive between common and albuminous nephritis: and that the alleged abnormal condition of the kidney is in some cases entirely unconnected with the supposed attendant alteration in the urine. After referring briefly to two cases, the one occurring in his own practice, the other in that of Dr. Lees, the author remarks: "while one of the preceding cases proves that we may have Bright's kidneys without albuminous urine, the other shows we may have albuminous urine without Bright's kidney: facts which, coupled together, militate strongly against the hypothesis, that the change in the structure of the kidney is connected with the appearance of albumen in the urine."—Dr. Graves, *Dublin Journal of Medical Science*, No. lx. *Jan.* 1842.

---

Non-identity of Smallpox and Cowpox. So long as the doctrine of the identity of smallpox and cowpox was put forward as an ingenious hypothesis, merely incapable of direct proof, but calculated to explain to the satisfaction of some persons the preservative powers of vaccination, the author was contented to let the matter rest; but when the experiments of Mr. Ceely and of his indefatigable imitators on the Continent and elsewhere, are triumphantly referred to in support of the doctrine, and when it has been recently recommended to some of our highest authorities to abolish the term vaccination altogether, and instruct the poor that cowpox and smallpox are identical, the author thinks it indispensably necessary to sift the matter thoroughly. After some remarks designed to explain Mr. Ceely's experiments in a different manner from that in which this gentleman himself interprets them, the author remarks:

The vaccine of man has its own peculiar characters. So has that of the cow. Human vaccine occasions no specific febrile disturbance, and consequently throws off no contagious emanations. It is frequently followed by a peculiar lichenous eruption. Before it has, by frequent transmissions, assimilated itself to the human constitution, it does not show its true characters, but frequently occasions ugly eruptions, which, in the early periods of vaccination, were often taken for smallpox, and have even been designated as such in later times by those who ought to have known better. Its areola is circular, and does not commence until the eighth day. It has the power of preserving the human body, to a certain extent, from the assaults of variola. It can only be received once by the human body in a perfect form. It can (after a certain time) be perpetuated from man to man in an uniform state of intensity. It can be produced in man by no combination of common causes. It is producible in man by inoculation alone. The characters of vaccinia in the cow are in several essential respects different. The vaccine disease in cows can be generated by common causes. It acknowledges in them both a local and a constitutional origin. We have no reason to believe that the passing through this disease gives to the cow immunity from any other disease. Whether it can be received twice by the cow, in a perfect form, I am not aware; nor whether it can be perpetuated from cow to cow in similar states of intensity. The differences between vaccine and variola are still greater. The variolous vesicle advances with areola from the very first. It cannot be perpetuated from man to man in similar states of intensity. Its reception into the human frame, by the mode of inoculation, is always attended by more or less fever. When received by the respiration, the febrile disturbance is often excessive, and may even, *per se*, prove fatal. It throws off, however, generated contagious emanations.

Dr. Baron has stated (Life of Jenner, vol. i. p. 245,) as a strong argument in favour of the theory of identity, " that it is impossible to doubt that some of the inferior animals have been liable to smallpox;" and he quotes, with approbation, Dr. Layard's conjecture that the *pestis bovilla* of 1780 was really smallpox. Now, if cows are subject to smallpox, how happens it that the inoculation of cows with variolous matter does not produce smallpox? We know that it does not; and we further know, that it produces vaccine: from all which I conclude, not that smallpox and cowpox are identical, but just the reverse, that they are antagonist affections.—Dr. Gregory, *Medical Gazette*, No. xi. *Dec.* 3, 1841.

---

Gallstones. The following are the general conclusions to which the author's researches have led him.

1st. That females are more liable to these affections than males: the proportion as three to one, exclusive of Dr. Heyfelder's cases, 2 to 1.

2d. That the greater number are persons of melancholic and bilious temperaments, and are in the middle and upper ranks of life.

3d. That mental disquietude is a very frequent cause.

4th. That sedentary habits and good living, especially eating, also excite these maladies, and that they are most frequent between the ages of thirty and sixty.

5th. That fat people are not, as most authors assert, more subject to the disease than those of spare habit.

6th. That the use of fermented drinks operates but little in the production of the complaint. This opinion is strengthened by the circumstance of gin-drinkers being, I believe, less liable to these affections; as well as their greater prevalence amongst females. In many of the cases the habits of the patients are not mentioned; but only two are described as intemperate.

7th. That these calculi may exist without producing but little, if any, inconvenience; and in the majority of cases there is no apparent structural change in the liver or gall-bladder.

In regard to treatment, the author, after noticing several methods in use, of the efficacy of which he appears to be sceptical, seems to confide principally in the salutary effects to be derived from air, exercise and regulation of diet, mental quiet, "stomachic" and alterative medicines, cold and tepid sponging, with frictions. He refers to Durande's celebrated remedy of which Dr. Copland speaks favorably, although the author distrusts Durande's own account of its success. The remedy consists of two parts of spirits of turpentine, and three of sulphuric ether, of which forty drops are to be taken every morning and during the passage of stones.—Mr. Crisp, *Lancet*, No. xi., *Dec.* 11, 1841.

---

Pseudo-morbid Appearances of the Brain and its Envelopes. By pseudo-morbid are meant those appearances which are not due to disease, but to physiological actions, which go on for some time after death; or which, at least, are very distantly and indirectly the consequence of the disease by which life is extinguished. The causes which operate in this way in the brain, giving rise to pseudo-morbid colorations and softening, are stated to be four: 1. Obstruction in the course of the venous circulation, occurring shortly before death. 2. Gravitation of the blood to the more dependent parts, the hyperemia by hypostasis of Andral. 3. Extravasal transudation or imbibition of the blood or of some of its component parts after death. 4. Spasmodic action of the muscular system, occurring towards the close of life.

The pseudo-morbid colorations are of two kinds, the spotted or the "injection sablée" of Lallemand, and the diffused or uniform; the former of which takes place chiefly in the medullary, the latter in the cortical substance. The former is the more frequent appearance, and much the more trustworthy as an indication of inflammation. It may result as a pseudo-morbid appearance from slow asphyxia, or from impediment to the circulation in the right side of the heart, or in the veins in the upper part of the body. It may happen from convulsions before closure of the fontanelles, or from muscular contractions in the body. When it occurs in particular parts of the brain only, or around an apoplectic effusion, the probability of the coloration being the result of true morbid action becomes strong. But on the *diffused* coloration, when surrounding apoplectic spots, the author is not disposed to place any reliance, as indicative of inflammatory action. Both *it* and a certain ramollissement, which often in such circumstances present themselves, being due to an absorbing action, which goes on in the substance of the brain for some time after death, and by which, more or less of the "extravasal exudation of the serum and colouring matter," are taken up. Orfila has seen pseudo-ramollissement, equally with the true, assume a variety of colours, as red, green, yellow, &c. Softening may be regarded as the result of *true* inflammatory action, when it is surrounded by a zone of red vessels, with small coagula, or when the softened portions are infiltrated with purulent matter.

In regard to the cerebral membranes, the author observes that there is one appearance very apt to be mistaken for actual disease or its result. This is opacity. But this appearance is in a great many cases due to simple distension of the membrane by subjacent fluid; on letting out which, the membrane itself is seen to be transparent. In like manner the cornea of either the living or dead eye may be made to look opaque, if distended by compression of the ball.—Dr. Patterson, *Edinburgh Medical and Surgical Journal*, No. 150, *Jan.* 1, 1842.

---

Paralysis from Visceral Disease. Ann Masters, ætat. 14, came to the Almshouse in 1837, with chronic diarrhœa, having a continual mucous discharge from the bowels. For this, many remedies had been used in vain. The diarrhœa continued obstinately, and she gradually became very weak in her lower extremities, and at length powerless and benumbed, being completely paralysed; the sphincters lost their power of acting, making her a loathsome and disgusting object; the mental functions were perfect; she still retained sensation in her arms, the use of her tongue, and all the members of the upper half of the body; she gradually wasted away, and at length died. The following were the appearances upon dissection: Body much emaciated; legs very thin. The stomach presented some patches of red upon the lining membrane. The colon was also somewhat inflamed, but the great seat of the disease was in the jejunum. The whole jejunum was in a state of high inflammation, the vessels being strongly injected. The mucous membrane was disorganized and pulpy. The spine was particularly examined, but no disease could be observed in the vertebræ, in the spinal marrow, or in the nerves of the lower extremity, except that they were greatly reduced in size. No morbid condition was observed in the great sympathetic nerve or ganglia. All the other viscera were sound.

This case clearly shows an enteritis, accompanied with paraplegia, and the only lesion found, was an inflammation of the intestines, the mental faculties being perfect, and the spinal marrow, vertebræ, and nerves being sound.—Dr. ZABRISKIE, *American Journal of the Medical Sciences*, No. iv., *Oct.* 1841.

---

PERFORATION OF THE STOMACH BY A WORM. A female child, eight years of age, after having been slightly unwell on the 19th of January, and relieved by some mild treatment, was again seized on the 30th, with vomiting of large quantities of dark-coloured fluid. The stomach could retain nothing, and the child died the following morning at five o'clock.

Externally, the body presented a healthy appearance; on cutting into the peritoneum, the intestines were found "completely covered" by a large quantity of bloody serum; on removing which the first thing which presented itself was a large worm (ascaris lumbricoides) lying upon the omentum. On prosecuting the examination, the author found an opening in the anterior surface of the stomach, about two inches from the pyloric extremity. The whole surface of the peritoneum was deeply injected, and of a vivid redness.—Mr. CHAMBERS, *Provincial Medical and Surgical Journal*, No. xxi. *Feb.* 19, 1842.

[Was not the real disease the common ulcerative perforation, the presence of the worm and its escape merely incidental?]

---

LEPROSY AND LEPER HOSPITALS. This is a continuation of the elaborate paper referred to in our last. The number of quaint references and quotations is immense, evincing the erudition and laborious research of the author. The principal point established in the present part is, that as regards its nosology, British leprosy, both of more early and more recent date, is identical with the elephantiasis græcorum.—Dr. SIMPSON, *Edinburgh Medical and Surgical Journal*, No. cl. *Jan.* 1, 1842.

